# Current Perspectives on Desmoid Tumors: The Mayo Clinic Approach

**DOI:** 10.3390/cancers3033143

**Published:** 2011-08-08

**Authors:** Siddharth B. Joglekar, Peter S. Rose, Franklin Sim, Scott Okuno, Ivy Petersen

**Affiliations:** 1 Musculoskeletal Oncology, Mayo Clinic, Rochester, MN55905, USA; E-Mail: joglekar.siddharth@mayo.edu; 2 Department of Orthopedics, Mayo Clinic, 200 1^st^ ST SW, Rochester, MN 55905, USA; E-Mail: rose.peter@mayo.edu; 3 Department of Oncology, Mayo Clinic, Rochester, MN55905, USA; E-Mail: okuno.scott@mayo.edu; 4 Department of Radiation Oncology, Mayo Clinic, Rochester, MN55905, USA; E-Mail: petersen.ivy@mayo.edu

**Keywords:** desmoids, fibromatosis, familial adenomatous polyposis, deep fibromatosis, aggressive fibromatosis

## Abstract

Desmoid tumors are a rare group of locally aggressive, non malignant tumors of fibroblastic origin that can lead to significant morbidity due to local invasion. Despite advances in the understanding of these tumors, their natural history is incompletely understood and the optimal treatment is still a matter of debate. Local control is the main goal of treatment and there has been a change in philosophy regarding the management of these tumors from aggressive surgical resection to function preservation. A multidisciplinary approach is essential to plan local control with acceptable morbidity. The current Mayo Clinic algorithm for the treatment of these tumors is based on institutional experience and the available evidence in the literature: asymptomatic/non progressive lesions away from vital structures are managed with observation and regular imaging; primary or recurrent desmoid tumors which are symptomatic or progressive or near vital structures are managed with wide surgical resection when wide surgical margins are possible with minimal functional and cosmetic loss. When positive or close surgical margins are likely, surgical resection with adjuvant radiotherapy or definitive radiotherapy is preferred. If likely functional or cosmetic deficit is unacceptable, radiotherapy is the treatment of choice. Unresectable lesions are considered for radiotherapy, chemotherapy or newer modalities however an unresectable lesion associated with a painful, functionless, infected extremity is managed with an amputation.

## 1. Introduction

Desmoid tumors are a rare group of locally aggressive, non malignant tumors of fibroblastic origin that can lead to significant morbidity due to local invasion and may even result in a fatal outcome when located around vital organs. Their clinical presentation, biological behavior and natural history can be quite varied and is incompletely understood at the present time. The optimal therapeutic approach depends on various factors, and a multidisciplinary approach is necessary to achieve local control with acceptable morbidity [1-3]. Despite progress in the understanding of these tumors and the treatment options, local recurrence remains a major problem. The current Mayo clinic approach to extraabdominal desmoid fibromatosis is summarized in light of the current knowledge regarding this tumor.

## 2. Epidemiology

**Figure 1.**
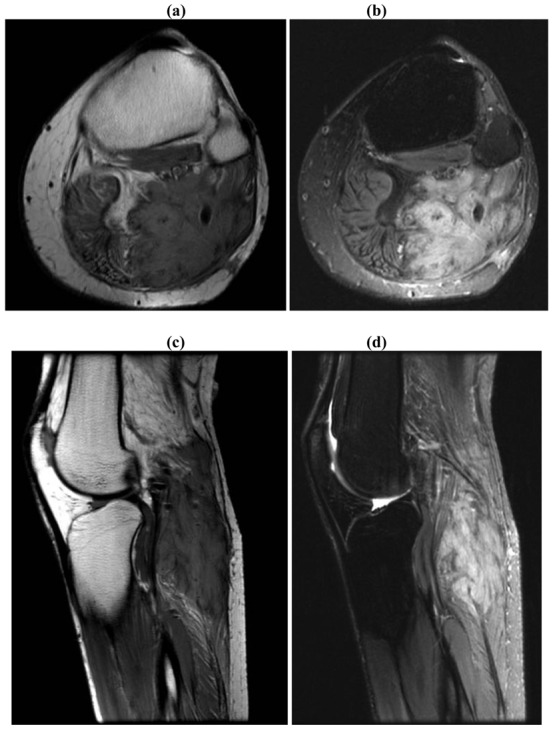
30/F with unresectable recurrence of desmoid tumor involving the posterior aspect of the left proximal calf. Initial tumor appeared during pregnancy and was resected following delivery. The recurrence was treated with close observation, discontinuation of oral contraceptives and sulindac. MRI findings: A large lobulated infiltrating mass with unusual architecture extending from just above the knee distally into the upper calf. The mass measures approximately 4.4 × 6.5 cm in axial diameters ×13.4 cm craniocaudal dimension. There are areas of relatively low T2 signal and avid gadolinium enhancement within the mass which could be seen with desmoid tumor. **(a)** Axial T1; **(b)** Axial T2; **(c)** Sagittal T1; **(d)** Sagittal T2.

These rare tumors have an annual incidence of two to four cases per million, with an average age at diagnosis of 40 years and a slight female predominance [4-6]. The exact etiology of these tumors is unknown, but hormonal, genetic, and physical factors all play a role in their development and growth. Although the majority of desmoid tumors are idiopathic, associations with estrogens, pregnancy and trauma have been documented in literature (Figure 1) [7,8].

**Figure 2.**
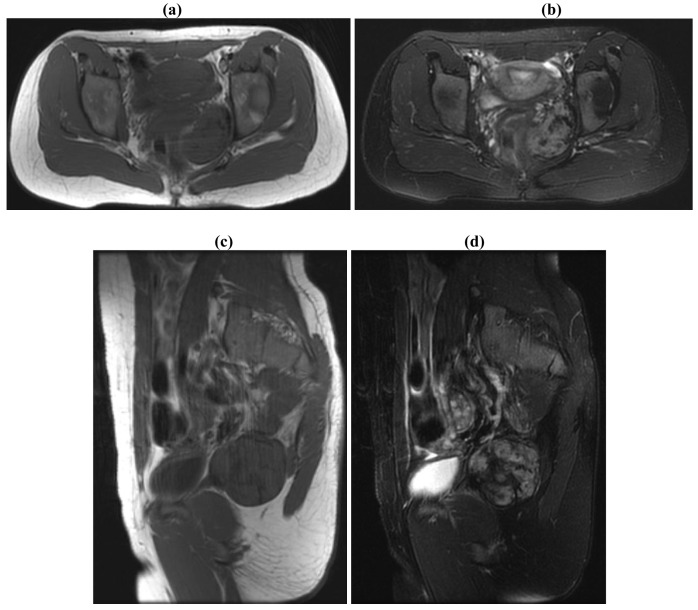
31/F with deep pelvic desmoid with a history of FAP treated with excision utilizing a combined Pfannenstiel and ischiorectal approach. Adjuvant radiation was administered as a result of positive margins. MRI Findings: There is an 8.4 × 6.8 × 5.5 cm enhancing irregularly-shaped mass centered in the left ischiorectal fossa. There is full-thickness invasion of the left obturator internus muscle. The mass extends across the midline to the right with invasion of the perirectal muscles as well as posteriorly up to, and likely involving a small portion of the gluteus maximus muscle on the left. **(a)** Axial T1; **(b)** Axial T2; **(c)** Sagittal T1; **(d)** Sagittal T2.

They may occur sporadically or in association with familial adenomatous polyposis (FAP). Approximately 7.5% of cases are associated with familial adenomatous polyposis (FAP) in the general population, while studies from a tertiary institution may see this rate being as high as 15% [6,9]. On the other hand 12–15% of patients with FAP develop desmoid tumors [10-12]. Desmoid tumors most commonly involve the extraabdominal locations in the general population whereas patients with FAP mostly present with intraabdominal disease (Figure 2) [5]. Mesenteric desmoid tumors are known to be the second leading cause of death in FAP patients [13,14]. There is inconsistency in the literature regarding the risk factors for desmoid tumors in FAP [15]. In general a positive family history, an APC mutation 3′ to codon 1399, previous abdominal surgery and the female sex have been implicated as risk factors in FAP [10,12].

The usual presentation is that of a slow growing mass without associated pain or discomfort. Depending on location the tumor may present with symptoms such as neurological deficit, joint stiffness or abdominal complaints. While desmoid tumors are known to spontaneously regress in a few cases, many continue to progress and need to be treated [16]. There is also evidence for varying periods of growth in the life of a lesion, including a stable phase [17].

## 3. Pathology

**Figure 3.**
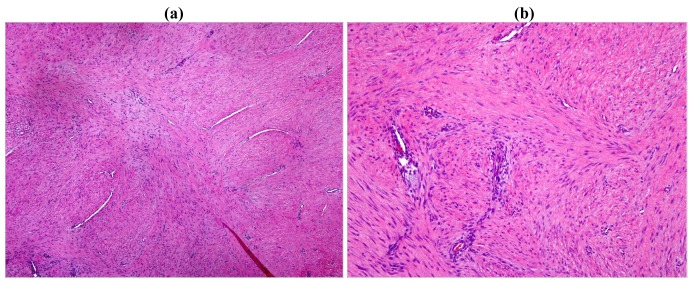
Typical histopathology pattern in low and high magnification for Desmoid Tumor. The lesion is poorly defined with infiltrative margins consisting of spindled fibroblasts arranged in broad sweeping fascicles separated by abundant collagen. The vessels, although thin-walled, are seen at scanning magnification. The nuclei of the proliferating lesion are lighter than those of the endothelial cells, and the smooth muscle cytoplasm in vessel walls is pinker than the surrounding myofibroblastic cytoplasm of the tumor cells. Mitotic figures are infrequent. **(a)** Low magnification; **(b)** High magnification.

**Figure 4.**
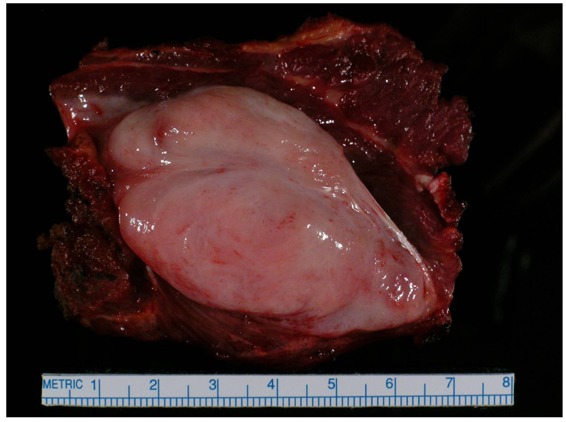
Gross specimen on cut section. These tumors occur as gray-white, firm, poorly demarcated masses. They are rubbery and tough, and infiltrate surrounding structures.

On gross examination, the tumors appear firm and the tumor usually extends beyond the pseudo-capsule. Microscopically, spindle-shaped cells are seen, separated by thick collagen fibers (Figures 3 and 4).

Immunohistochemistry is positive for vimentin and smooth muscle actin, but negative for desmin, cytokeratin, and S-100. Molecular studies of X-chromosome inactivation have demonstrated that these tumors are a monoclonal proliferation of cells and not a reactive process as thought in the past [18]. Somatic mutations in the beta-catenin (CTNNB1) gene have been shown to occur with high frequency (98%) in sporadic desmoid tumors. Furthermore it has been shown that certain CTNNB1 (45-F) mutations are at particular risk for recurrence [19]. The beta-catenin gene (CTNNB1) has been also associated with hyperplastic wound healing responses [20]. Desmoid tumors associated with familial adenomatous polyposis (FAP) have been shown to be associated with mutations in the adenomatous polyposis coli (APC) gene [11]. Both CTNNB1 and APC are part of the Wnt signaling pathway and mutations in either gene result in stabilization of the beta-catenin protein leading to activation of the T-cell factor/lymphoid enhancer factor (TCF/Lef) family of transcription factors. This molecular biological trait may be targeted for therapies in the future [20]. Recent research has shown that activity of these tumors may be modulated through a combination of receptors with tyrosine kinase activity, platelet derived growth factor receptors A and B (PDGFR) and/or Cyclooxygenase-2 (COX-2) overexpression. Consequently there has been interest in newer therapies such as Tyrosine kinase inhibitors and COX-2 inhibitors [21-23].

## 4. Management

Desmoid tumors are histologically benign tumors that do not metastasize. However they can be locally aggressive and hence the main goal of desmoid treatment is local control. Extraabdominal tumors are not commonly associated with mortality however morbidity and disfigurement may be related to treatment or tumor progression. The literature lacks level I evidence in the form of randomized controlled trials to compare the relative efficacy various treatment modalities. A multitude of treatment options are available and choosing the appropriate method for achieving local control depends on the functional and cosmetic outcomes of each method and the associated complications [1,24].

## 5. Nonsurgical Management

The natural history of desmoids tumors continues to be an enigma. Desmoids have been reported to remain stable for prolonged periods of time or even regress spontaneously in some reports [17,25-27]. The surgical challenges and morbidity associated with the treatment of desmoid tumors have forced a change in global trends toward adopting a pro-conservative approach. A “wait and watch” approach has been promoted by several recent studies [27]. An improved understanding of the molecular biology of these tumors may also help in identifying patients at risk for recurrence [19,28]. Adjuvant treatment modalities may be utilized early in these high risk cases.

### 5.1. Expectant Management

The infiltrative and recurrent nature of desmoid tumors can render surgical resection challenging if acceptable function and cosmesis is to be maintained. Aggressive attempts at resection have the potential to make the treatment worse than the disease. Treatment in each case has to be individualized with multidisciplinary participation [1]. Acceptable levels of function and cosmesis will vary from patient to patient depending on location, occupation, handedness, prior level of functioning and morbidity caused by the disease itself. It is a determination that is finally made by the patient in concert with the health care providers. The plastic surgery team, occupational therapists, physical medicine and rehabilitation specialists and members from the amputee service can provide valuable input to help with this decision. In order to avoid the morbidity of surgery or radiotherapy, a period of watchful waiting may be the most appropriate management in selected patients [1,25,27]. The current trend is strongly in favor of treating asymptomatic desmoids with observation, only reserving treatment for those tumors that may pose danger to vital structures or show continued growth [1,16,25-27,29].

### 5.2. Systemic Therapy

Systemic therapy is an option in unresectable or recurrent disease. Available options include hormonal therapies, nonsteroidal anti-inflammatory drugs (NSAIDs), interferon, and chemotherapy. The use of hormonal therapy for the treatment of these tumors is based on the association of these tumors with pregnancy or contraceptives pills and reports of regression after menopause or oophorectomy [30-33]. Success rates of around 50% have been obtained with hormonal treatments and other agents such as NSAIDs, Vit C, and warfarin [34-38]. The most common regimen uses high dose tamoxifen at 120 mg per day along with sulindac. Response and control rates have been reported to be over 50% [39]. Some patients cannot tolerate this treatment or fail to respond. Such patients with symptomatic, progressive disease who can tolerate chemotherapy can be managed with either low-dose or standard antisarcoma chemotherapy. The use of low-dose methotrexate and vinorelbine for up to one year has been shown to control desmoids with manageable side effects. More aggressive standard antisarcoma systemic therapy with doxorubicin- or ifosfamide-based chemotherapy is also effective in desmoids. Although it is unclear what the optimal regimen is, patients appear to have quicker responses to the standard antisarcoma therapy especially with regimens containing doxorubicin [39-41]. The use of chemotherapy has to be weighed against the potential for morbidity. The use of tyrosine kinase imatinib has shown some moderate control of desmoids [42]. The recent positive reports, of sorafenib in desmoid will need to be confirmed by other groups [43]. Tamoxifen an antagonist of the estrogen receptor is currently being studied for effectiveness in hormone receptor positive breast desmoid tumors. Newer research suggests that deregulation of the mammalian target of rapamycin (mTOR) cell proliferation/survival pathway may play an important role in desmoid tumor biology especially when the APC/β-catenin pathway is disrupted. Several randomized controlled trials are currently recruiting patients to study the role of these newer systemic treatment modalities for Desmoid tumors [44].

### 5.3. Radiotherapy

Retrospective studies indicate that radiation therapy may improve the local control of desmoid tumors, in both the adjuvant and the primary setting [4,45-48]. A review of literature concluded that the rates of local control with either surgery with radiotherapy or radiotherapy alone are significantly better than surgery alone regardless of the margins achieved at surgery [49]. The relative superiority of radiotherapy alone or combined with surgical management is amplified in cases with positive margins. This underscores the importance of combined modality management in tumors with positive margins and that of radiotherapy alone in tumors which are unresectable [50,51]. This does come at the cost of short term and long term radiotherapy related complications which are observed in 17% of patients especially with radiation doses higher than 56Gy [52]. The best results are associated with high dose radiation and it may take up to two years for the tumor to regress [46,53]. The rate of local recurrence associated with radiotherapy is significantly increased when radiotherapy is used as the sole modality in doses less than 50 Gy. No differences in the rate of local recurrence are observed with high or low dose radiotherapy when combined modality management is utilized [49]. The most common complications observed with the use of radiotherapy are fibrosis, paresthesias, edema, fractures and local skin irritation [49].

## 6. Surgery

Surgical resection is the primary treatment modality for desmoids tumors when functionally and cosmetically acceptable with reported local control rates of 75–80% [54,55]. It is also recommended when the tumor is close to vital structures and progression would be associated with high morbidity or mortality [13,14,56]. Despite the reported high local control rates with surgery alone, local recurrence rates have varied from *24*–7*7%* between series [4,26,54,55,57-59]. Although margins are thought to influence local control in soft tissue sarcoma, the literature on desmoid tumors presents conflicting evidence. While some studies have stressed on the futility of aggressive surgery to obtain negative margins, other studies have shown that margins do influence local recurrence rates [2,54,55,58-61]. A recent comparative analysis of the available literature concluded that wide surgical margins significantly influence recurrence in surgically treated desmoid tumors [62]. Contrarian opinions regarding this issue; sometimes from the same institutions; underlines the importance of issues such as selection bias and study design in retrospective studies [2,54,58,60,63]. No randomized controlled trials are available as yet to guide treatment. Surgery is also recommended in recurrent cases whenever feasible since local control rates are similar to primary surgical excision [7,58]. In the case of patients with unresectable extremity desmoids tumors function preserving procedures should be the goal. In case of patients who have failed systemic therapy and/or radiation and whose only option is amputation it may be possible to safely follow them unless the limb is painful, functionless or infected [59].

## 7. Conclusions

Desmoid tumors are locally aggressive fibrous tissue tumors with a tendency for local recurrence despite adequate surgical resection. Asymptomatic or non-progressive tumors may be carefully observed. Surgical resection is favored when functionally and cosmetically acceptable however combined modality management is useful in recurrent or unresectable tumors. Radiation may be indicated after margin positive resection or if unresectable with impending functional problems. Systemic therapies should also be considered in cases where the tumor is unresectable, especially in cases where radiation toxicity may be also unacceptable.

**Figure 5.**
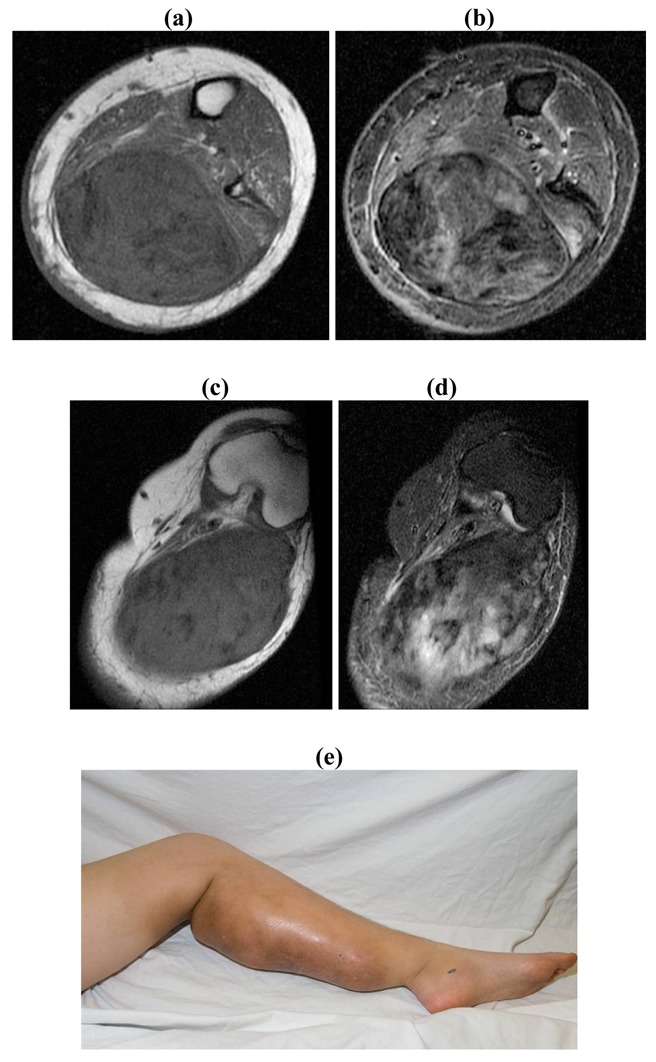
23/F with a four year history of failed non-operative treatment of unresectable symptomatic desmoid tumor involving the left lower extremity. Patient had continued to progress despite 6000cGy of radiation and chemotherapy. Patient had 15 degrees of knee flexion contracture and 20 degrees of ankle flexion contracture in the setting of a painful leg. Patient was treated with an above knee amputation. MRI Findings: A huge heterogeneous mass is seen in the posterior compartment of the left thigh, extending superiorly above the knee joint within the biceps femoris and measuring 8 × 10.7 cm The popliteal vessels and tibial nerve are displaced due to the enlarging mass and abnormal T2 signal extends from the mass to these displaced neurovascular structures which could represent peritumoral edema or microscopic infiltration. There is encasement of the common fibular nerve in addition. **(a)** Axial T1 at level of leg; **(b)** Axial T2 at level of leg; **(c)** Axial T1 at level of knee; **(d)** Axial T2 at level of knee; **(e)** Clinical photo.

The Mayo Clinic Algorithm: there has been a change in the philosophy regarding the management of desmoid tumors since the review by Pritchard *et al* was published in 1996 [4]. In the original review only 12% of the tumors were managed nonsurgically. The emphasis in the current literature has shifted to nonoperative management in case of asymptomatic and stable lesions to avoid the morbidity associated with aggressive surgical resection. The current Mayo Clinic approach to desmoid tumors reflects these trends:

- Asymptomatic/non progressive/Away from vital structures: Observation with regular imaging
- Primary/Recurrent Desmoid - Symptomatic/Progressive/Near vital structures:
  - Wide surgical margins possible with minimal functional and cosmetic loss: Surgical resection
  - Positive or close surgical margins: Surgical resection with adjuvant radiotherapy/Radiotherapy
  - Functional/Cosmetic loss unacceptable: Radiotherapy
  - Unresectable: Radiotherapy/Chemotherapy/Newer modalities
  - Unresectable associated with painful/ functionless/infected extremity: Amputation (Figure 5)
